# Transcription yield of fully 2′-modified RNA can be increased by the addition of thermostabilizing mutations to T7 RNA polymerase mutants

**DOI:** 10.1093/nar/gkv734

**Published:** 2015-07-24

**Authors:** Adam J. Meyer, Daniel J. Garry, Bradley Hall, Michelle M. Byrom, Hannah G. McDonald, Xu Yang, Y. Whitney Yin, Andrew D. Ellington

**Affiliations:** 1 Institute for Cellular and Molecular Biology, University of Texas at Austin, Austin, TX 78712, USA; 2 Altermune Technologies, LLC, Irvine, CA 92606, USA; 3 Department of Pharmacology and Toxicology, Sealy Center for Structural Biology, University of Texas Medical Branch, Galveston, TX 77555, USA; 4 Department of Chemistry and Biochemistry, University of Texas at Austin, Austin, TX 78712, USA; 5 Center for Systems & Synthetic Biology, University of Texas at Austin, Austin, TX 78712, USA

## Abstract

On average, mutations are deleterious to proteins. Mutations conferring new function to a protein often come at the expense of protein folding or stability, reducing overall activity. Over the years, a panel of T7 RNA polymerases have been designed or evolved to accept nucleotides with modified ribose moieties. These modified RNAs have proven useful, especially *in vivo*, but the transcriptional yields tend to be quite low. Here we show that mutations previously shown to increase the thermal tolerance of T7 RNA polymerase can increase the activity of mutants with expanded substrate range. The resulting polymerase mutants can be used to generate 2′*-O-*methyl modified RNA with yields much higher than enzymes currently employed.

## INTRODUCTION

RNA is widely versatile and useful, but its chemical instability can render it unsuitable for many therapeutic and biotechnology functions. Oligonucleotides with altered chemistry, especially modifications of 2′ position of the (deoxy)ribose have proven to be of great value (1). 2′*-O-*methyl RNA has a greater *T*_m_, faster hybridization kinetics and greater stability as antisense probes (2), and siRNA with 2′-fluoro and 2′*-O-*methyl RNA have also proven to be less immunogenic, more stable in serum and more target-specific (3–7). Additionally, *in vitro* selection with 2′ modified NTPs has yielded aptamers and ribozymes with greater stability (8–13).

While modified RNA can be chemically synthesized, it is often preferable to enzymatically produce it (especially for *in vitro* selection) (14). T7 RNA polymerase has long been utilized for the generation of RNA *in vitro*, and has previously been engineered and evolved to have an expanded substrate range. Most famously, the Y639F mutant allows for the polymerization of RNA transcripts containing nucleotides with 2′-fluoro and 2′-amino modified ribose (15–17). A further mutation, H784A, is thought to eliminate premature termination following the incorporation of a modified nucleotide, and the Y639F, H784A (‘FA,’ Supplementary Table S1) double mutant can incorporate nucleotides with bulky modifications at the 2′ position (e.g. 2′*-O-*methyl)(18,19).

We have previously taken a directed evolution approach, in which the aforementioned Y639 and H784 residues, as well as the important R425 and G542 were randomized (20). The resulting library was enriched for T7 RNA polymerase variants that retained the ability to transcribe RNA *in vivo* (with natural ribose) and then screened for altered substrate specificities *in vitro*. A mutant, termed ‘RGVG,’ (R425, G542, Y639V, H784G plus additional E593G and V685A mutations that arose during the selection) showed strong activity with 2′*-O-*methyl UTP. A second mutant, termed ‘VRS,’ (G542V and H784S as well as the additional H772R mutation) was able to incorporate 2′-fluoro modified pyrimidines despite remaining wild-type at the important Y639 position.

More recent works have also uncovered the ‘2P16’ mutant (Supplementary Table S1), which is reported to have enhanced activity over its RGVG parent in terms of 2′*-O-*methyl NTP incorporation (21). Additionally the R425C mutant (Supplementary Table S1) is reported to enable the creation of fully 2′*-O-*methyl RNA (22).

Collectively, the above described mutants represent the best options for the incorporation of nucleotides with 2′-modifed ribose moieties. While the unique catalytic properties of these enzymes make them useful tools, several of them suffer from low activity, even with normal ribonucleotides. It has been proposed that mutations that confer new activity in an enzyme also destabilize the protein, rendering it less active overall (23–27). In this work, we apply this idea by adding mutations known to increase the *T*_m_ of T7 RNA polymerase to several mutants with altered nucleotide specificity. We show that thermostabilizing mutations in T7 RNA polymerase are able to act orthogonally and in an additive way to the substrate specificity mutations, rescuing low activity without impacting substrate preference.

## MATERIALS AND METHODS

### Preparation of T7 RNA polymerase variants

The T7 RNA polymerase ORF was cloned into pQE-80L (Qiagen). All T7 RNA polymerase variants were derived from this plasmid either by Mega-primer polymerase chain reaction (28) or Isothermal assembly (29). Plasmids were transformed into BL21-gold (Agilent) *E. coli* cells. Cells were grown in 2xYT media at 37°C overnight. Subcultures were grown at 37°C until reaching OD600 ∼0.7–0.8 at which point 1 mM IPTG was added. Cells were grown 4 h at 37°C, pelleted and frozen at −80°C. Pellets were resuspended in binding buffer (50 mM Tris-HCl pH 8.0, 0.5 M NaCl, 5 mM imidazole). Resuspended cells were lysed via sonication on ice using 50% probe amplitude for 3 min (1 s on, 1 s off). Cell debris was pelleted by centrifugation for 30 min at 10 000 g. His-tagged T7 RNA polymerase was purified by immobilized metal affinity chromatography (IMAC). The lysate was run over 1 ml (bead volume) Ni-NTA (Fisher) gravity column pre-equilibrated with binding buffer. The column was washed with 10 column volumes of wash buffer (50 mM Tris-HCl pH 8.0, 0.5 M NaCl, 20 mM imidazole). T7 RNA polymerase was eluted from the column by the addition of 3 column volumes of elution buffer (50 mM Tris-HCl pH 8.0, 0.5 M NaCl, 250 mM imidazole). Dialysis was performed in final storage buffer (50 mM Tris-HCl pH 8.0, 100 mM NaCl, 1 mM DDT, 1 mM EDTA). Dialates were adjusted to 1 mg/ml and added to an equal volume of glycerol (final concentration 0.5 mg/ml).

### Crystallization of the transcribing thermostable ‘M5’ RNA polymerase initiation complex

Crystals were obtained using hanging-drop method at 20°C, the drops contain 300 μM T7 RNA polymerase M5 variant, 350 μM partial duplex promoter DNA (3′-ATTATGCTGAGTGACCCTCT/5′-TAATACGACTCACT), 4 mM each of GTP and UTP, and the well solution contains 20% PEG 8000, 200 mM (NH_4_)_2_SO_4_, 0.25% β-octyl glucopyranoside, 20% glycerol and 100 mM Tris-HCl pH 8–8.5. The crystals were flash frozen directly in liquid nitrogen, and diffraction data were collected using a synchrotron source. Data were processed using program HKL, and the structures were determined by molecular replacement method using wide-type T7 RNA polymerase initiation complex as a search model (pdb: 1qn). The structure was refined using program PHENIX to an *R*_factor_ = 19.8% and *R*_free_ = 23.6%.

### *In vitro* transcription assays

Real-time transcription reactions contained 40 mM Tris-HCl pH 8.0, 30 mM MgCl_2_, 6 mM spermidine, 6 mM each NTP (or modified NTP), 10 mM DTT, 500 nM T7 RNA polymerase, 500 nM DNA template and 0.17 mg/ml DFHBI (in DMSO). Reactions were incubated for up to 4 h at 37°C with Spinach fluorescence (Excitation 469 nm / Emission 501 nm) reading taken 1–4 min in a Safire monochromator (Tecan). Spinach templates were made by thermal cycling overlapping primers (5′-AATATAATACGACTCACTATAGAGGAGACTGAAATGGTGAAGGACGGGTCCAGTGCTTCG and 5′-GAAAAGACTAGTTACGGAGCTCACACTCTACTCAACAGTGCCGAAGCACTGGACCCG) with Accuprime Pfx in its standard buffer (94°C:2 min, 12 cycles [94°C:15 s, 50°C:30 s, 68°C:30 s], 68°C:1 min). Templates were purified by QIAquick Gel Extraction Kit (Qiagen).

End point transcription reactions contained 40 mM Tris-HCl pH 8.0, 30 mM MgCl_2_, 6 mM spermidine, 6 mM each NTP (or modified NTP), 10 mM DTT, 500 nM T7 RNA polymerase, 500 nM DNA template. Reactions were incubated for up to 4 or 20 h at 37°C. DNA templates were made as above. Reactions containing rVmU (2′*-O-*methyluridine) or rRmY (2′*-O-*methylpyrimidines) were run 4 h; the transcripts were labeled by inclusion of 0.17 μM (α^32^P)ATP (3000 Ci/mMol), and analyzed by denaturing PAGE. rGmH (2′*-O-*methyladenosine and 2′*-O-*methylpyrimidines) reactions were run 20 h, labeled by inclusion of 0.17 μM (α^32^P)GTP (3000 Ci/mMol), and analyzed by denaturing PAGE. RGVG-M6 reactions were run 20 h, incubated for 1 h at 37°C with 0.03 U/μl Baseline-ZERO DNase in its supplied buffer, analyzed by denaturing PAGE and imaged after staining in SYBR-Gold. The buffer comparison used the buffers listed in the figure, was run 20 h, incubated for 1 h 37°C with 0.03 U/μl Baseline-ZERO DNase in its supplied buffer, analyzed by denaturing PAGE and imaged after staining in SYBR-Gold.

mN (2′*-O-*methylnucleotides) incorporation was performed in the permissive buffer, which contained 200 mM HEPES pH 7.5, 5.5 mM MgCl_2_, 2 mM spermidine, 0.5 mM each 2′*-O-*methyl-NTP, 40 mM DTT, 0.01% Triton, 10% PEG8000, 1.5 mM MnCl_2_, 10 U/ml yeast inorganic pyrophosphatase, 200 nM RNA polymerase and 200 nM DNA. Reactions were run 20 h, incubated for 1 h 37°C with 0.03 U/μl Baseline-ZERO DNase in its supplied buffer, analyzed by denaturing PAGE and imaged after staining in SYBR-Gold.

^32^P polyacrylamide gels were exposed to a storage phosphor screen (Molecular Dynamics) before imaging on a STORM 840 Phospoimager (GE Healthcare). Autoradiographs were analyzed using ImageQuant (GE Healthcare).

### Thermal melt measurements

The relative thermal stability of each T7 RNA polymerase was assessed by incubating 0.5 mg/ml enzyme in PBS buffer with TexasRed dye (Invitrogen). Enzyme/dye mixtures were equilibrated at 37°C for 10 min and heated at a rate of 0.07°C/s to 97°C using a LightCycler 96 thermocycler, while fluorescence was monitored (Excitation 577 nm / Emission 620 nm). The first derivatives of the change in fluorescence as a function of time were used to approximate the relative *T*_m._ Data were analyzed using Roche thermocycler software.

### RNA quantification

Reactions were performed in the permissive buffer, which contained 200 mM HEPES pH 7.5, 5.5 mM MgCl_2_, 2 mM spermidine, 0.5 mM each nucleotide, 40 mM DTT, 0.01% Triton, 10% PEG8000, 1.5 mM MnCl_2_, 10 U/ml yeast inorganic pyrophosphatase, 200 nM RNA polymerase and 320 nM DNA. Reactions were run 20 h, incubated for 30 min at 37°C with 0.03 U/μl Baseline-ZERO DNase in its supplied buffer, and stopped with 50 mM EDTA. Reactions were purified by denaturing PAGE. Briefly, reactions were mixed with an equal volume 2x denaturing dye, run on a 9% polyacrylamide gel, cut out and eluted with 0.3 M NaOAc in 1x TE by freezing at −80°C and incubating at 37°C overnight. The eluate was ethanol precipitated, resuspended in water and quantified on a NanoDrop 2000 spectrophotometer.

## RESULTS

### Stabilizing substitutions increase the activity the T7 RNA polymerase G542V H784S variant

A previous selection for RNA polymerases with altered substrate specificity (20) focused on altering four amino acids proximal to the incoming nucleotide (30,31). One of the selected variants (G542V, H772R, H784S; ‘VRS’) could incorporate 2′-F-modified pyrimidines. VRS had mutations at two of the randomized residues (*i.e*. G542V and H784S). Interestingly, an H772R mutation also arose during the selection, although position 772 had not originally been being randomized. H772R is not near the substrate recognition domain, but has also arisen in other selections for T7 RNA polymerase activity (32,33). This led us to hypothesize that H772R is a mutation that generally stabilizes the structure of the polymerase, and that it suppressed instabilities that accrued due to the introduction of G542V and / or H784S.

**Figure 1.**
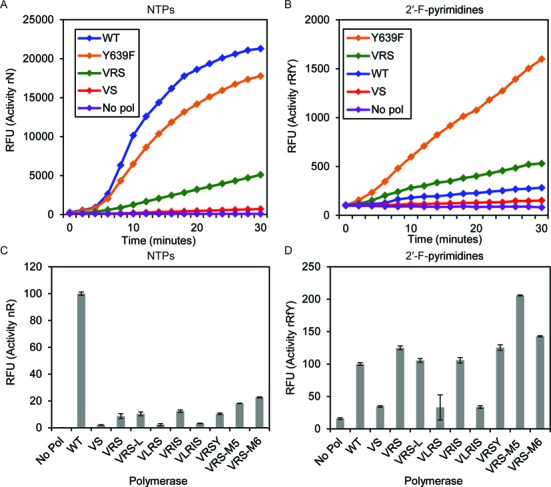
Stabilizing mutations increase the activity of the VRS mutant. **(A)** Real time measurement of ribonucleotide (rN) transcriptional output. **(B)** Real time measurement of 2′-fluoropyrimidine (rRfY) transcriptional output. **(C)** Measurement of ribonucleotide (rN) transcriptional output after 3 h. **(D)** Measurement of 2′-fluoropyrimidine (rRfY) transcriptional output after 3 h. Fluorescent readings (in Relative Fluorescent Units, RFU) indicate the presence of the fluorescent aptamer, spinach. Data shown in panels (A) and (B) result from single experiments. Data shown in panels (C) and (D) are the average of three independently assembled reactions with error bars representing standard error.

To test this hypothesis, we constructed a derivative of VRS without H772R, a variant we termed ‘VS’. We then assayed purified enzymes for their ability to polymerize RNA composed either of natural NTPs (rN) or of ribo-purines and 2′-F-pyrimidines (rRfY; Figure 1A, B). Real-time polymerase activity was assayed by transcribing the fluorescent aptamer Spinach in the presence of DFHBI (34). Spinach will bind DFHBI and fluoresce irrespective of whether it is transcribed as a purely ribo-aptamer or when substituted with 2′-F-pyrimidines, although the 2′-F-pyrimidine version is only about 30% as fluorescent as the purely ribonucleotide version. 2′*-O-*methyl substituted Spinach is not detectably fluorescent. As predicted, VS showed a decreased activity for each substrate composition assayed.

### Additional substitutions further increase the activity of T7 RNA polymerases at higher temperatures

The finding that putative structural stabilizer could abet substrate specificity variants led us to expand upon the original hypothesis and create additional variants that might further stabilize the structure and enhance the function of T7 RNA polymerase. One or more substitutions that were hypothesized to increase the stability of T7 RNA polymerase were introduced into VRS. Some of these mutations have been described previously and have been shown to increase thermal stability (S430P, N433T, S633P, F849I and F880Y) (35) or promoter clearance (P226L) (36) of T7 RNA polymerase. Computational modeling had indicated that other mutations (V625L and V783I) would allow T7 RNA polymerase to pack more efficiently (Ben Borgo and James Havranek, personal communications). These mutations had also arisen in parallel in a ‘neutral drift’ selection for T7 RNA polymerase in which stabilizing mutations are expected to accumulate (37,38).

Mutations that were primarily from the patent literature (35), the so-called ‘M5’ (S430P, N433T, S633P, F849I and F880Y) and ‘M6’ (P266L, S430P, N433T, S633P, F849I and F880Y) mutations increased the activity of VRS for both rN and rRfY incorporation (Figure 1C, D). The ‘M5’ mutations arose from selections of T7 RNA polymerase with transcriptional activity at higher temperatures, and we confirmed that the M5 polymerase exhibits transcription activity similar to the wild-type enzyme at 37°C, but transcribes 10-fold more RNA than the wild-type at 50°C.

### Thermostabilizing substitutions further increase the activity of T7 RNA polymerases with altered substrate specificities

**Figure 2.**
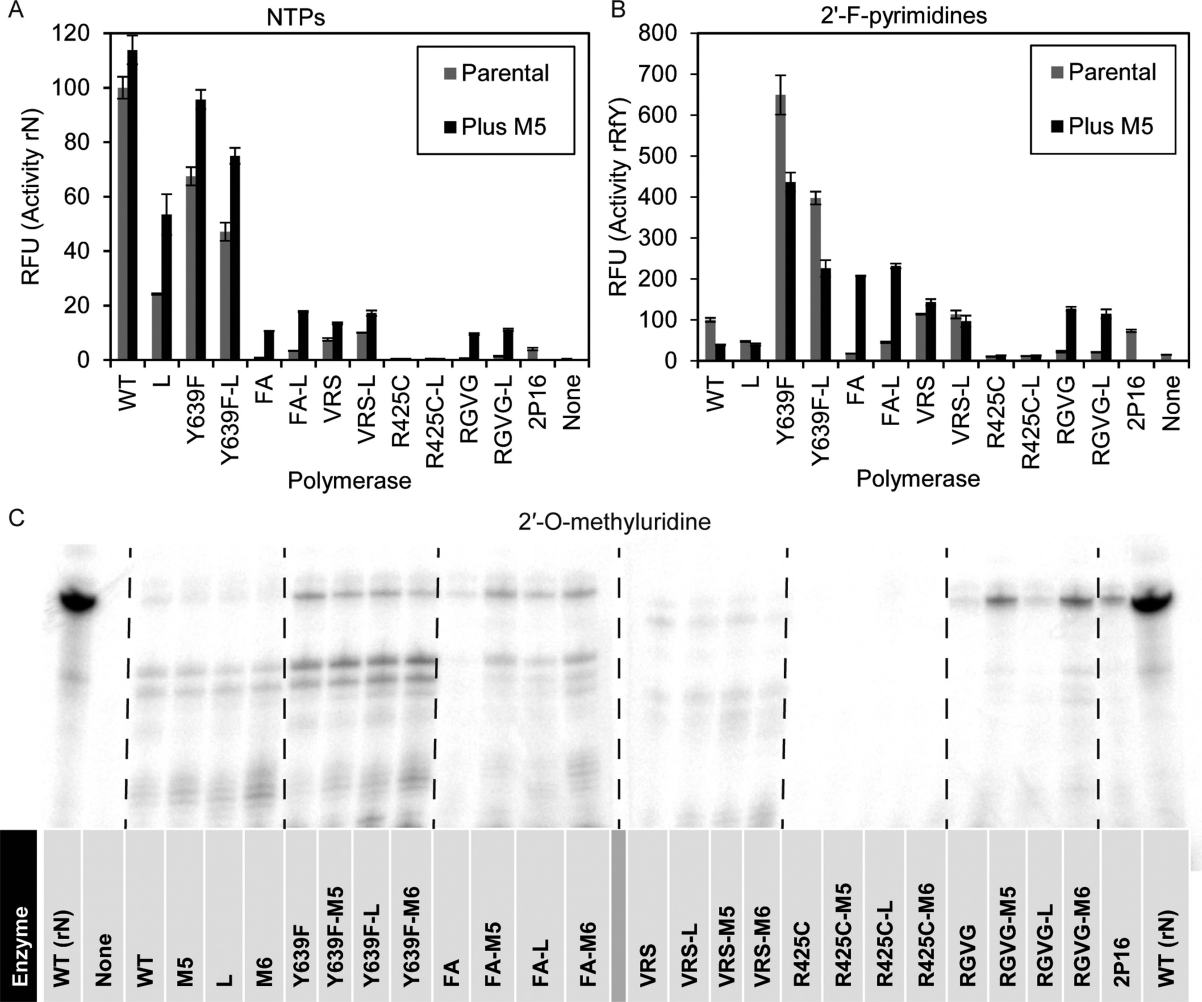
Stabilizing mutations increase the activity of the several T7 RNA polymerase substrate specificity mutants. **(A)** Measurement of ribonucleotide (rN) transcriptional output after 1 h. **(B)** Measurement of 2′-fluoropyrimidine (rRfY) transcriptional output after 2 h. Fluorescent readings (in Relative Fluorescent Units, RFU) indicate the presence of the fluorescent aptamer, spinach. Error bars represent standard error resulting from three independently assembled reactions. **(C)** Transcription assay for incorporation of 2′*-O-*methyluridine (rVmU). Transcripts were labeled by inclusion of (α^32^P)ATP and analyzed by denaturing PAGE. A reaction of WT T7 RNA polymerase with ribonucleotides (rN) is included for comparison. Transcriptions ran 4 h, two distinct gels are shown. The data shown are taken from a single experiment.

Given that stabilizing mutations appeared to improve the performance of T7 RNA polymerase variants with altered substrate specificities, we further hypothesized that the addition of M5 and M6 mutations to a wider variety of substrate specificity variants might yield polymerases that could robustly incorporate modified nucleotides. Starting from several known polymerases that could differentially incorporate ribose modifications (WT, Y639F, FA, RGVG, VRS and R425C) the P266L, M5 and M6 (which is M5 plus P266L) substitutions were added (Supplementary Table S1). We further included a recently described mutant, 2P16 (I119V, G225S, K333N, D366N, F400L, E593G, Y639V, S661G, V685A, H784G, F880Y) that was uncovered in a high-throughput screen for increased activity with NTPs (21). These 25 polymerases were purified and assayed for transcriptional activity *in vitro* (Figure 2).

RNA molecules containing 2′-F-pyrimidines and 2′*-O-*methylnucleotides are commonly used for medical and biotechnology applications (1), thus we investigated the ability of different specificity variants (WT, Y639F, FA, RGVG, VRS and R425C) combined with the M5 and M6 mutations to incorporate modified nucleotides. Each polymerase was tested for its ability to transcribe RNA composed of natural nucleotides (rN; Figure 2A), purines and 2′-F-pyrimidines (rRfY; Figure 2B), or purines, cytidine, and 2′*-O-*methyluridine (rVmU; Figure 2C). We failed to detect any transcription from the R425C family of specificity variants, despite reports that R425C alone can transcribe fully 2′*-O-*methyl RNA (22). Wild-type T7 RNA polymerase and Y639F activities with natural nucleotides was slightly increased by the M5 mutations, but were not better when challenged with unnatural nucleotides. The FA (Y639F, H784A) and RGVG (E593G, Y639V, V685A, H784G) variants, which are the two most commonly used enzymes for the transcription of 2′*-O-*methyl RNA, each demonstrated increased activity with each nucleotide composition upon addition of the M5 or M6 mutations. While 2P16 is indeed more active than its parent, RGVG (as previously claimed (21)), it is not as active as either RGVG-M5 or RGVG-M6 (Figure 2).

**Figure 3.**
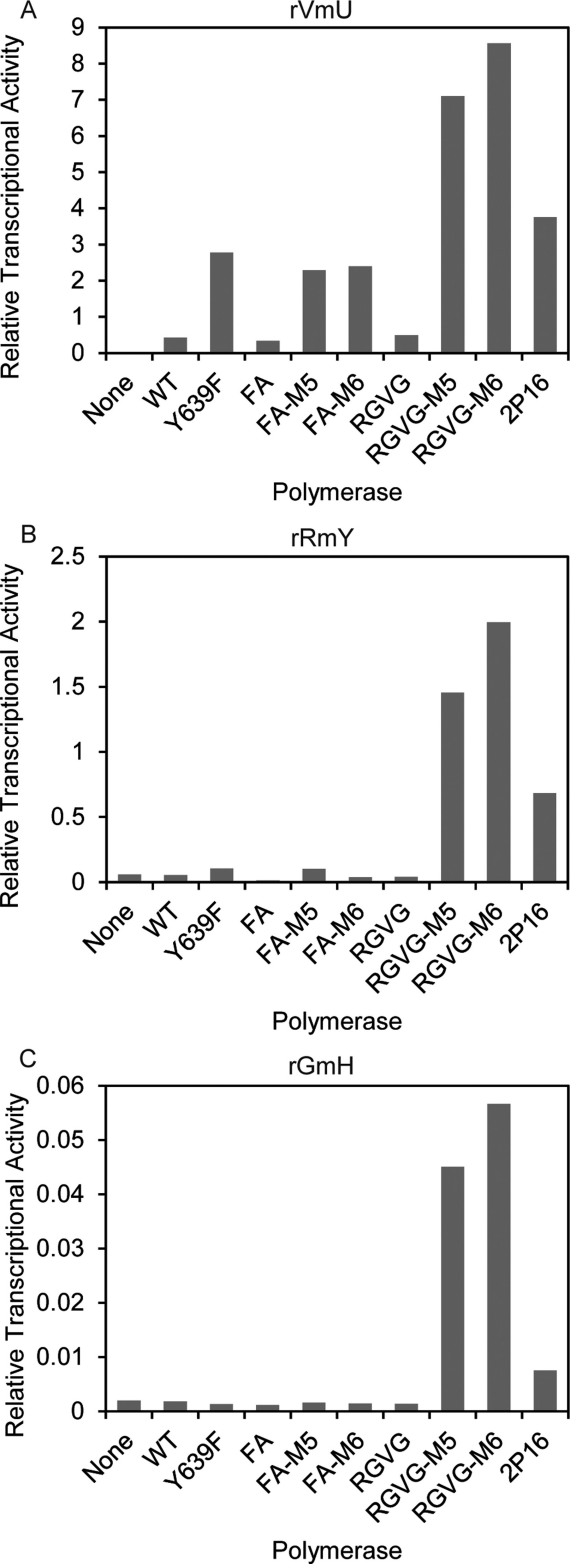
Stabilized T7 RNA polymerase mutants have increased yield of heavily modified RNAs. Transcription assay for incorporation of 2′*-O-*methyluridine (rVmU; **A**), 2′*-O-*methylpyrimidines (rRmY; **B**), or 2′*-O-*methyladenosine and 2′*-O-*methylpyrimidines (rGmH; **C**). Transcripts were labeled by inclusion of (α^32^P)ATP (rVmU and rRmY) or (α^32^P)GTP (rGmH) and analyzed by denaturing PAGE. All values are normalized to 100, representing the yield of WT T7 RNA polymerase with ribonucleotides (rN). Transcriptions ran 4 h (rVmU and rRmY) or 20 h (rGmH).

Based on the above results, FA-M5, FA-M6, RGVG-M5 and RGVG-M6 are clear improvements over the enzymes currently used for the incorporation of nucleotides with bulky 2′-modifications (FA and RGVG). We therefore sought to also determine the extent to which they could transcribe RNA of increasingly diverse nucleotide compositions. We assayed a subset of the most active polymerases for the ability to incorporate purines, cytidine, and 2′*-O-*methyluridine (rVmU), purines and 2′*-O-*methylpyrimidines (rRmY), and guanosine, 2′*-O-*methyladenosine and 2′*-O-*methylpyrimidines (rGmH) (Figure 3, Supplementary Figures S1–S3). As was the case for rN and rRfY above (Figure 2A, B), the M5 and M6 mutations enhanced the activity of the FA and RGVG enzymes for each set of nucleotide composition. RGVG-M6 was the most active enzyme in all conditions, yielding at least 25-fold more RNA than the FA mutant, which is the most commonly used enzyme for generating 2′*-O-*methyl RNA.

### The M5 mutations increase the thermal stability of substrate specificity mutants

**Figure 4.**
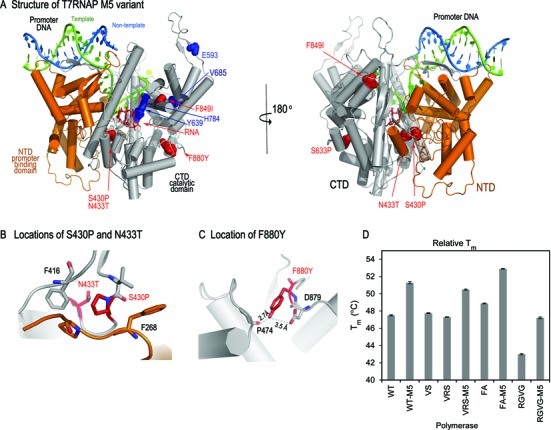
The M5 mutations and the thermal stability of T7 RNA polymerase mutants. **(A**) Crystal structure of T7 RNA polymerase M5 variant initiation complex that contains an NTD (orange) and a CTD (gray), a partial duplex promoter DNA consists of a 23-nt template (green) and 17-nt non-template (blue) and a 3-nt RNA (red). M5 mutations are shown in red spheres, and RGVG mutations shown in blue spheres. **(B)** The local environment of the S430P and N433T mutations. S430P enhances hydrophobic interaction with F268 and F416, **(C)** F880Y generated new hydrogen-bond with backbone of P474 and D879. **(D)** Thermal melt assays were performed for several mutants T7 RNA polymerase. First derivatives of the change in fluorescence as a function of time were used to approximate the relative *T*_m_. Data shown are the average of three independently assembled reactions with error bars representing standard error.

To better understand the structural basis for this improved activity, we crystallized and determined structure the M5 protein (Figure 4, Supplementary Table S2). T7 RNA polymerase consists of an N-terminal promoter binding domain (NTD) and a C-terminal catalytic domain (CTD) (Figure 4A). All five mutations in the M5 protein are located in the C-terminal catalytic domain. Two of them, S430P and N433T are located in the interface of NTD and CTD, S430P substitution enhances hydrophobic interaction with F268 and F416, thereby increase domain interaction (Figure 4B). Scanning microcalorimetry measurement showed that the *T*_m_ for CTD alone is 7°C lower than in the intact protein, strongly suggesting the CTD is stabilized by inter-domain interaction with the NTD (39). F880Y substitution generates hydrogen-bonding interactions with P474 and D879, thereby increasing internal stability of the CTD (Figure 4C). Although introduction of M5 substitution cause no gross conformational changes from the wild-type (30) (the superposition of the backbones of the two proteins yields an RMSD = 0.512 Å), these substitutions strengthen inter-domain interaction that is important for protein stability, as well as enhancing CTD intradomain strength. This explains that the F880Y mutation alone is insufficient to increase VRS activity (see VRSY in Figure 1C and D).

The RGVG mutations are also located in the CTD: E593G and V685A are located in the fingers domain that is responsible for template DNA binding (Figure 4A), and perhaps for DNA unwinding to provide a single-stranded template for RNA synthesis. Y639V and H784G are in the active site and directly contribute to the catalysis of phosphodiester bond formation between the incoming nucleotide and the 3′-end of the nascent RNA. In the wild-type T7 RNA polymerase, H784 protrudes into the minor-groove of the DNA:RNA hybrid, ensuring correct base-pairing interactions. The hydroxyl group of Y639 forms a H-bonding interaction with the 2′-OH of ribose, and substitution with a valine should yield a more promiscuous polymerase.

Thermal-melt assays indicate that, for all T7 RNA polymerase variants tested, addition of the M5 mutations increased their thermal stability (Figure 4D). The weakly active RGVG mutant has a *T*_m_ almost 5°C lower than that of WT T7 RNA polymerase, but this loss of stability, and RGVG's activity are rescued by the M5 mutations. Contrary to our expectations, however, the similarly weak VRS and FA mutants do not have low melting temperatures, and the H772R mutation did not have the expected effect on *T*_m_. It seems that the increase in activity due to the addition of these mutations cannot be solely attributed to an increase of stability. However, it is nonetheless true that the M5 mutations increase the activity of all substrate specificity mutants tested.

### T7 RNA polymerase RGVG-M6 is effective for high-yield transcription of fully-modified RNA

**Figure 5.**
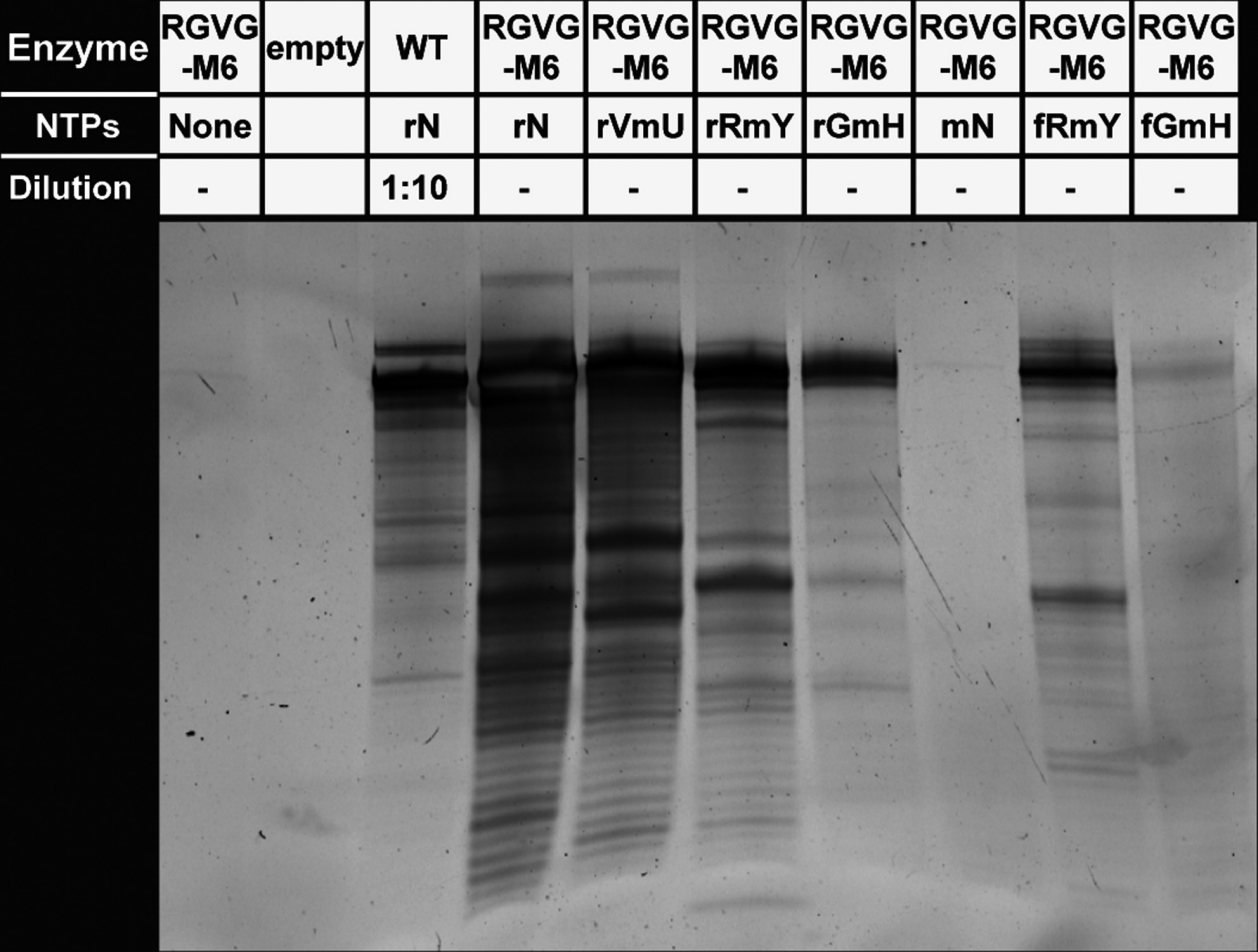
RGVG M6 can transcribe fully-modified RNA. Transcription assay for RGVG-M6 catalyzed incorporation of ribonucleotides (rN); 2′*-O-*methyluridine (rVmU); 2′*-O-*methylpyrimidines (rRmY); 2′*-O-*methyladenosine and 2′*-O-*methylpyrimidines (rGmH); 2′*-O-*methylnucleotides (mN); 2′-fluoro-purines and 2′*-O-*methylpyrimidines (fRmY); and 2′-fluoro-guanosine, 2′*-O-*methyladenosine and 2′*-O-*methylpyrimidines (fGmH). Transcripts were analyzed by denaturing PAGE and imaged after staining in SYBR-Gold. Transcriptions ran 20 h. A reaction (10-fold diluted) containing WT T7 RNA polymerase with ribonucleotides (rN) is shown for comparison.

As the 2′-hydroxyl group of ribose renders RNA unstable for many biological applications, the generation of fully-modified RNA is desirable. After demonstrating that RGVG-M6 could catalyze the formation of RNA containing three 2′*-O-*methylnucleotides, we assayed its ability to generate fully-modified RNA. RGVG-M6 was able to polymerize a combination of 2′-F-purines and 2′*-O-*methylpyrimidines (fRmY) as well as a combination of 2′-F-guanosine, 2′*-O-*methyladenosine and 2′*-O-*methylpyrimidines (fGmH). However, fully 2′*-O-*methyl RNA (mN) was not obtained under these conditions (Figure 5).

**Figure 6.**
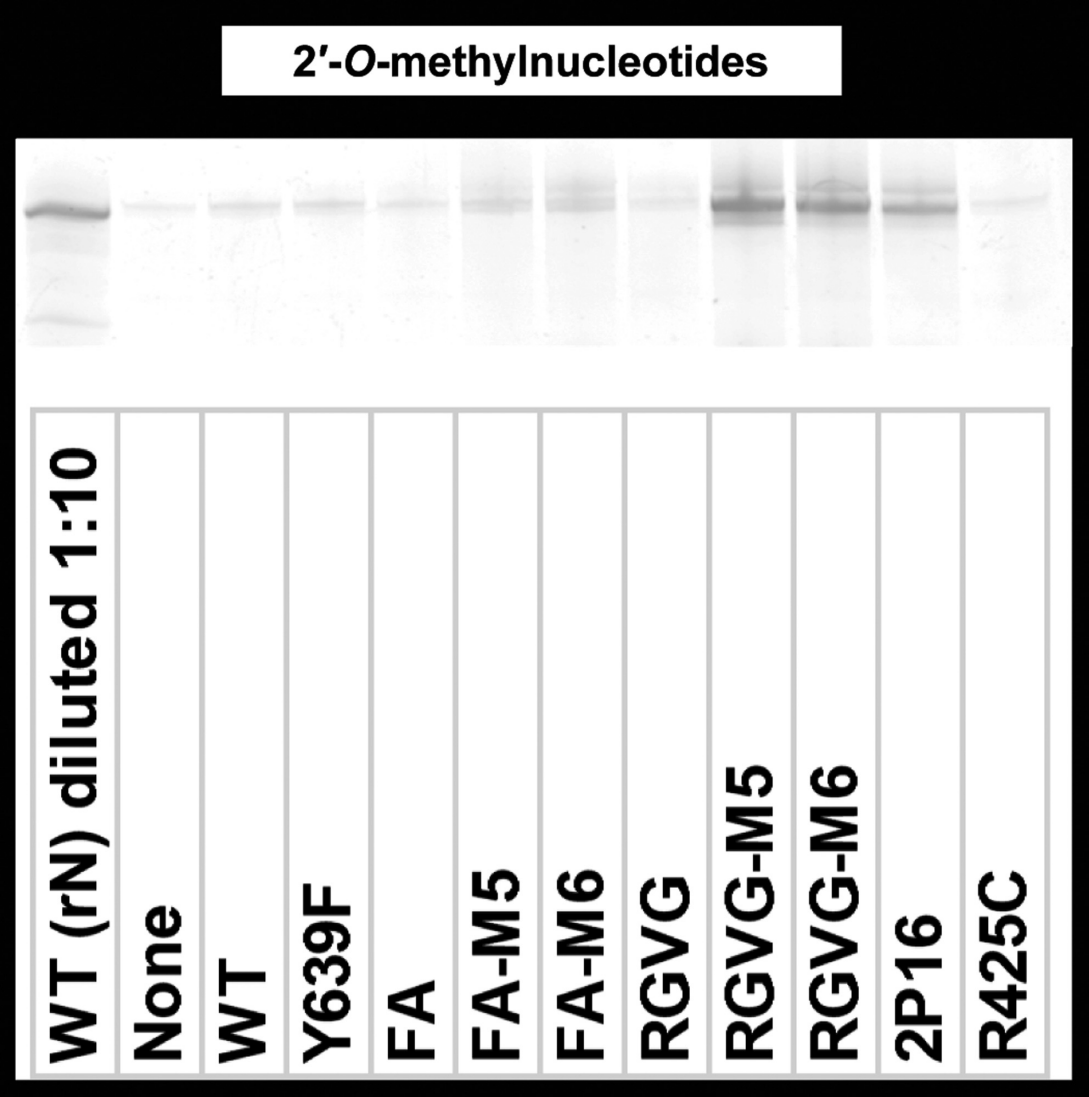
Transcription assay for incorporation of 2′*-O-*methylnucleotides (mN) in a permissive buffer. Transcripts were analyzed by denaturing PAGE and imaged after staining in SYBR-Gold. Transcriptions ran 20 h. A reaction (diluted 10-fold) containing WT T7 RNA polymerase with ribonucleotides (rN) is shown for comparison.

Previous reports of 2′*-O-*methylnucleotide incorporation have used more permissive buffer compositions that include manganese as well as rGMP and/or rGTP (12). We tested RGVG-M6's ability to polymerize 2′*-O-*methylnucleotides in several such permissive buffers (Supplementary Figure S4), and identified a buffer that is compatible with generating fully 2′*-O-*methyl RNA without including rGMP or rGTP. This buffer composition (200 mM HEPES pH 7.5, 5.5 mM MgCl_2_, 2 mM spermidine, 0.5 mM each 2′*-O-*methyl-NTP, 40 mM DTT, 0.01% Triton, 10% PEG8000, 1.5 mM MnCl_2_, 10 U/ml yeast inorganic pyrophosphatase, 200 nM RNA polymerase and 200 nM DNA) has previously been reported to be compatible with 2′*-O-*methylnucleotide polymerization (12), and optimizes both divalent cation composition and molecular crowding effects. RGVG-M5 and RGVG-M6 generate substantially more RNA in this buffer than other polymerases tested (Figure 6) including the commercially relevant enzyme Y639L H784A (Supplementary Figure S5) (40). While this buffer has been optimized for transcription by FA and RGVG-M6, it remains possible that 2P16, R425C and Y639L H784A would have higher activity in another buffer.

In order to demonstrate RGVG-M6's ability to generate large quantities of modified RNA for the purposes of *in vitro* selection, we performed several reactions simulating the generation of RNA pools. Transcriptions were performed, RNA was gel purified and RNA was quantified by spectrophotometry (Table 1). From transcriptions of the Spinach aptamer by RGVG-M6, we were able to recover 4.6 molecules of 2′*-O-*methyl (mN) RNA, or 5.5 molecules of 2′-F-guanosine, 2′*-O-*methyladenosine, 2′*-O-*methylpyrimidines (fGmH) RNA per molecule of DNA template. This is about 40% as efficient as transcription of the Spinach aptamer with NTPs by wild type T7 RNA polymerase, which yielded 12.4 molecules of RNA per molecule of DNA template. Transcription of an N42 aptamer pool by RGVG-M6 led to the recovery of 5.5 molecules of mN RNA or 4.9 molecules fGmH RNA per input template. This is nearly 70% as efficient as transcription of an N42 aptamer pool with NTPs by wild type T7 RNA polymerase, which yielded 7.7 molecules of RNA per molecule of DNA template. It should be noted, however, that the wild type T7 RNA polymerase can yield about 20-fold more rN RNA in a standard commercial buffer than in the permissive buffer.

**Table 1.** Quantification of modified RNA yields by RGVG-M6

Polymerase	Nucleotide composition	Template	Yield (transcripts per template)
WT	rN	Spinach	12.4
RGVG-M6	mN	Spinach	4.6
RGVG-M6	fGmH	Spinach	5.5
WT	rN	N42	7.7
RGVG-M6	mN	N42	5.5
RGVG-M6	fGmH	N42	4.9

## DISCUSSION

As engineered proteins explore sequence/fitness landscapes there is an inherent trade-off between activity and stability (23,25). As enzyme engineers endeavor to create proteins further removed from the wild-type, they are often confronted by these physical limitations. In this work we show that including globally stabilizing mutations in T7 RNA polymerase assists with accommodating mutations that alter substrate specificity. The VRS mutant, capable of incorporating 2′-F-pyrimidines, became roughly two-fold more active upon the addition of the M5 or M6 mutations, while retaining its initial altered substrate specificity. Similarly, the FA and RGVG mutants, capable of incorporating 2′*-O-*methyl NTPs, became at least 10-fold more active upon the addition of the M5 or M6 mutations. The greatly improved activity of the mutants RGVG-M5 and RGVG-M6 ultimately allows them to polymerize 2′-*O-*methyl RNA more effectively than any other known enzyme. They are capable of generating transcripts containing 2′-fluoro and/or 2′*-O-*methyl NTPs in high yields and can polymerize nucleic acid consisting entirely of modified nucleotides. These enzymes should prove useful in creating modified nucleic acids for *in vitro* selection (11,41) as well as for therapeutic applications (1,6,8).

This work further highlights the importance of phenotypic additivity or synergy. That is, protein variants conferring different phenotypes can potentially be combined to generate new variants with multiple phenotypes. The combined mutations can potentially interfere with one another, act independently, or even synergize and potentially support one another. In this case, mutations conferring altered substrate specificity to T7 RNA polymerase rendered it less active overall. Mutations known to increase the thermal stability of T7 RNA polymerase are able to increase overall activity without otherwise impacting substrate specificity. The idea that stabilizing mutations may be needed to uncover new functionality has been explored previously (24–27). For example, mutations to TEM-1 β-lactamase that provide resistance to third-generation cephalosporins also weaken its overall activity. This loss of activity can be overcome by the addition of the M182T mutation, a global stabilizer that arises in directed evolution studies as well as the clinic (23). What is particularly curious about our results is that the mechanistic basis of global stabilization was not immediately obvious: the repair of thermal stability was apparent in one instance (RGVG) but not in two others (VRS and FA), and no overt structural differences were imparted by the stabilizing mutations. This highlights the notion that the stabilizing mutations may have multiple roles beyond resisting temperature challenges, and thereby improve protein evolution by moving the proteins to new areas of fitness landscapes that are generally more active or robust to mutation. Even in the absence of a mechanistic explanation, though, this principle is of great engineering value: irrespective of whether stabilizing mutations are arrived at by evolutionary drift or selection (38,42) or by design (herein), they can potentiate new phenotypes in proteins.

Since premature termination, especially in initially transcribed sequences (ITS), remains a major problem for unnatural nucleotide polymerization (36), the impact of the P266L substitution (the additional substitution in M6) was assessed. P266L tends to reduce overall RNA yield, and has been associated with enhanced promoter clearance and fewer abortive products. However, compared to the M5 variants, the M6 variants have similar yields of full length RNA. While P266L thus appears not to be additive or synergistic with stabilizing and substrate specificity mutations, it nonetheless may still prove beneficial in some contexts. The choice of whether to use an M5 or M6 variant will ultimately depend on the DNA template, nucleotide composition, and intended use of the RNA product.

## Supplementary Material

SUPPLEMENTARY DATA
