# Supplementary material for: Transcription yield of fully 2′-modified RNA can be increased by the addition of thermostabilizing mutations to T7 RNA polymerase mutants
Source: Nucleic Acids Res. 2015 Jul 24;43(15):7480–8. doi: 10.1093/nar/gkv734 (PMC4551944; doi:10.1093/nar/gkv734)
Supplement: SUPPLEMENTARY DATA [file supp_43_15_7480__index.html]

Transcription yield of fully 2′-modified RNA can be increased by the addition of thermostabilizing mutations to T7 RNA polymerase mutants — SUPPLEMENTARY DATA 

# Transcription yield of fully 2′-modified RNA can be increased by the addition of thermostabilizing mutations to T7 RNA polymerase mutants

## SUPPLEMENTARY DATA

- SUPPLEMENTARY DATA
