# Supplementary material for: Transcription yield of fully 2′-modified RNA can be increased by the addition of thermostabilizing mutations to T7 RNA polymerase mutants
Source: Nucleic Acids Res. 2015 Jul 24;43(15):7480–8. doi: 10.1093/nar/gkv734 (PMC4551944; doi:10.1093/nar/gkv734)
Supplement: SUPPLEMENTARY DATA [file supp_gkv734_nar-02604-y-2014-File008.docx]

**Supplementary Tables and Figures**

**Table S1. List of T7 RNA polymerase mutants**

**
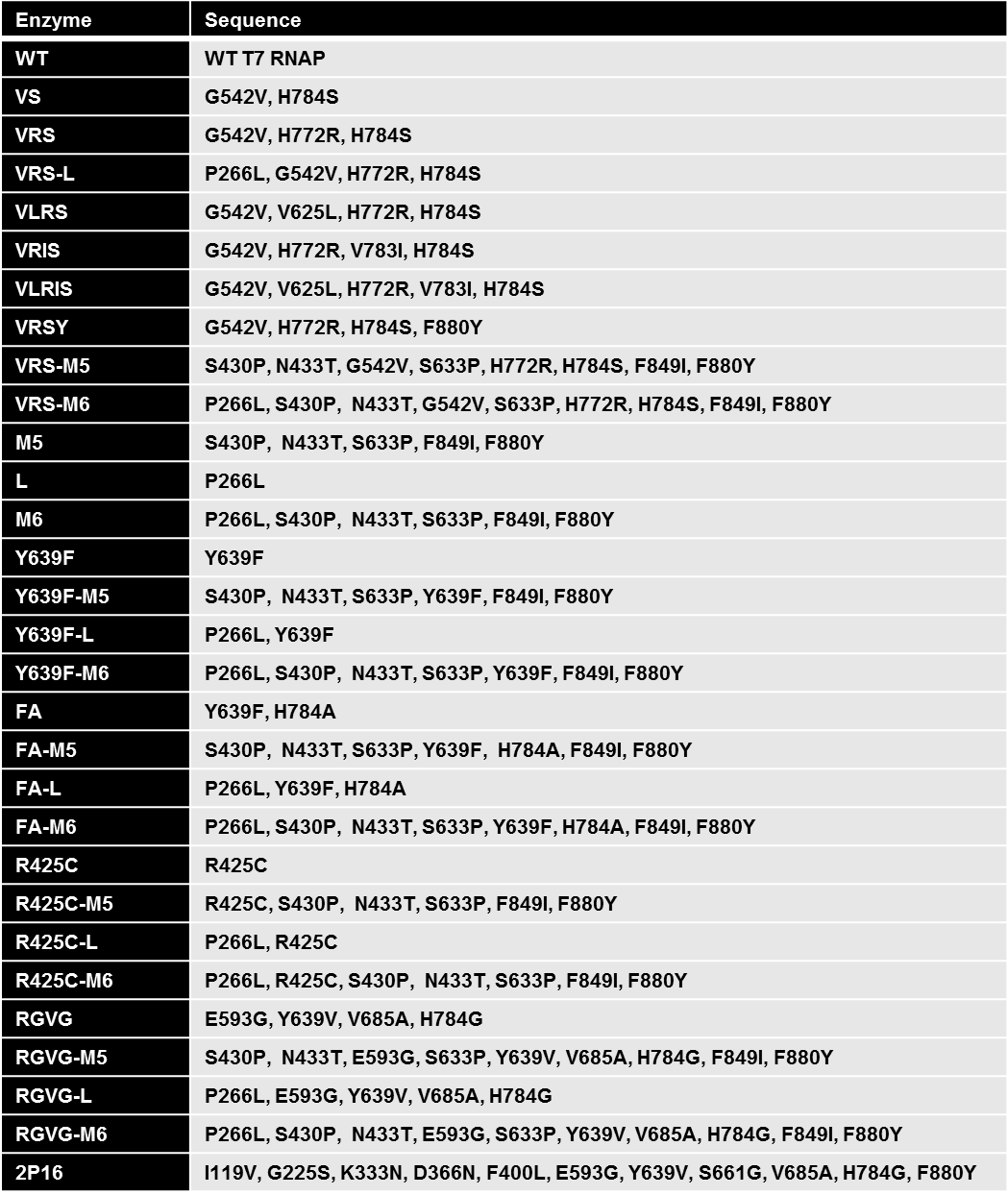
**

**Figure S1. Transcription assay for incorporation of 2′-*O*-methyluridine (rVmU). Transcripts were labelled by inclusion of (α^32^P)ATP and analyzed by denaturing PAGE. Transcriptions ran four hours. A reaction containing WT T7 RNA polymerase with ribonucleotides (rN) is shown for comparison.**

**
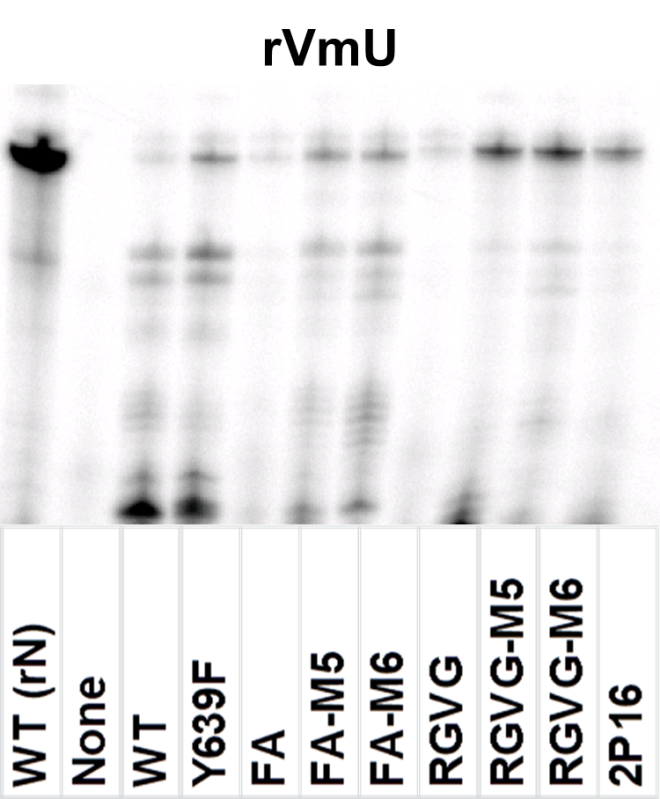
**

**Figure S2. Transcription assay for incorporation of 2′-*O*-methylpyrimidines (rRmY). Transcripts were labelled by inclusion of (α^32^P)ATP and analyzed by denaturing PAGE. Transcriptions ran four hours. A reaction containing WT T7 RNA polymerase with ribonucleotides (rN) is shown for comparison.**

**
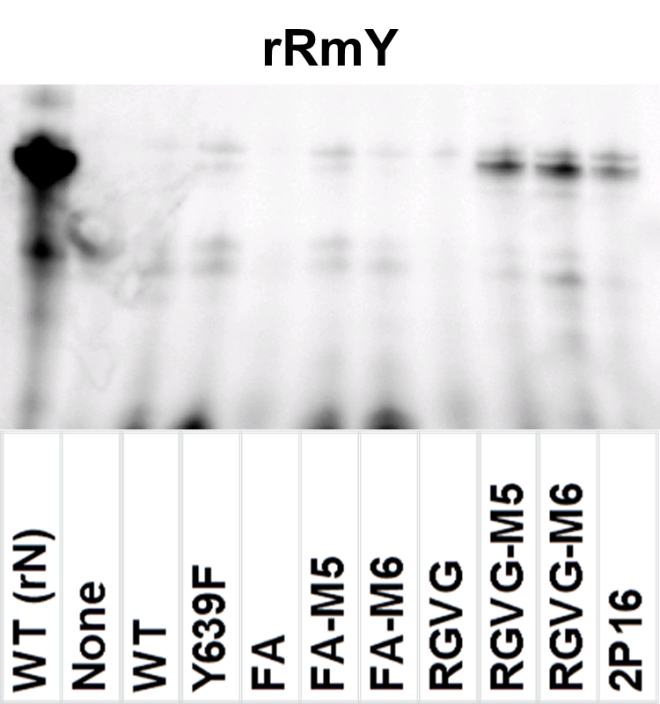
**

**Figure S3. Transcription assay for incorporation of 2′-*O*-methyladenosine and 2′-*O*-methylpyrimidines (rGmH). Transcripts were labelled by inclusion of (α^32^P)GTP and analyzed by denaturing PAGE. Transcriptions ran 20 hours. A reaction (diluted 50-fold) containing WT T7 RNA polymerase with ribonucleotides (rN) is shown for comparison.**

**
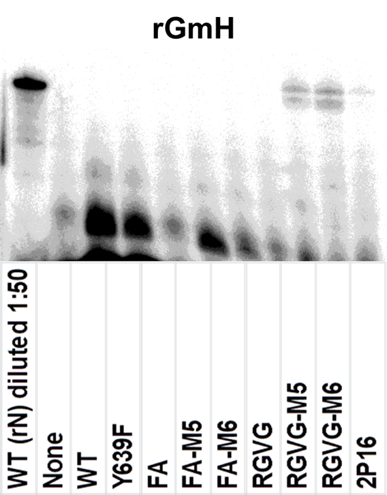
**

**Table S2. Crystallographic data and refinement statistics**

| Space group | **P 2_1_ 2_1_ 2** |
| --- | --- |
| Unit cell | **a=219.854 b=75.685 c=80 α=β=γ=90°** |
| Unique reflections | **30741 (2953)** |
| Multiplicity | **9.5** |
| Completeness (%) | **95.09 (83.18)** |
| Wilson B-factor | **50.18** |
| R-merge | **0.08** |
| Reflections used for R-free | **0.08** |
| R-work | **0.2322 (0.3213)** |
| R-free | **0.2660 (0.4598)** |
| Number of non-hydrogen atoms | **7735** |
| Macromolecules | **7610** |
| Protein residues | **902** |
| Nucleic acids residues | **32** |
| Water | **93** |
| RMS(bonds) | **0.004** |
| RMS(angles) | **0.78** |
| Ramachandran favored (%) | **93** |
| Ramachandran outliers (%) | **1** |
| Average B-factor  macromolecules  ligands  solvent | **53.40**  **53.20**  **122.20**  **43.60** |

**Figure S4. RGVG-M6 transcription of 2′-*O*-methylnucleotides (mN) in various buffers. Transcripts were analyzed by denaturing PAGE and imaged after staining in SYBR-Gold. Transcriptions ran 20 hours. A reaction containing RGVG-M6 with ribonucleotides (rN) is shown for comparison. The composition of each reaction is shown below.**

**
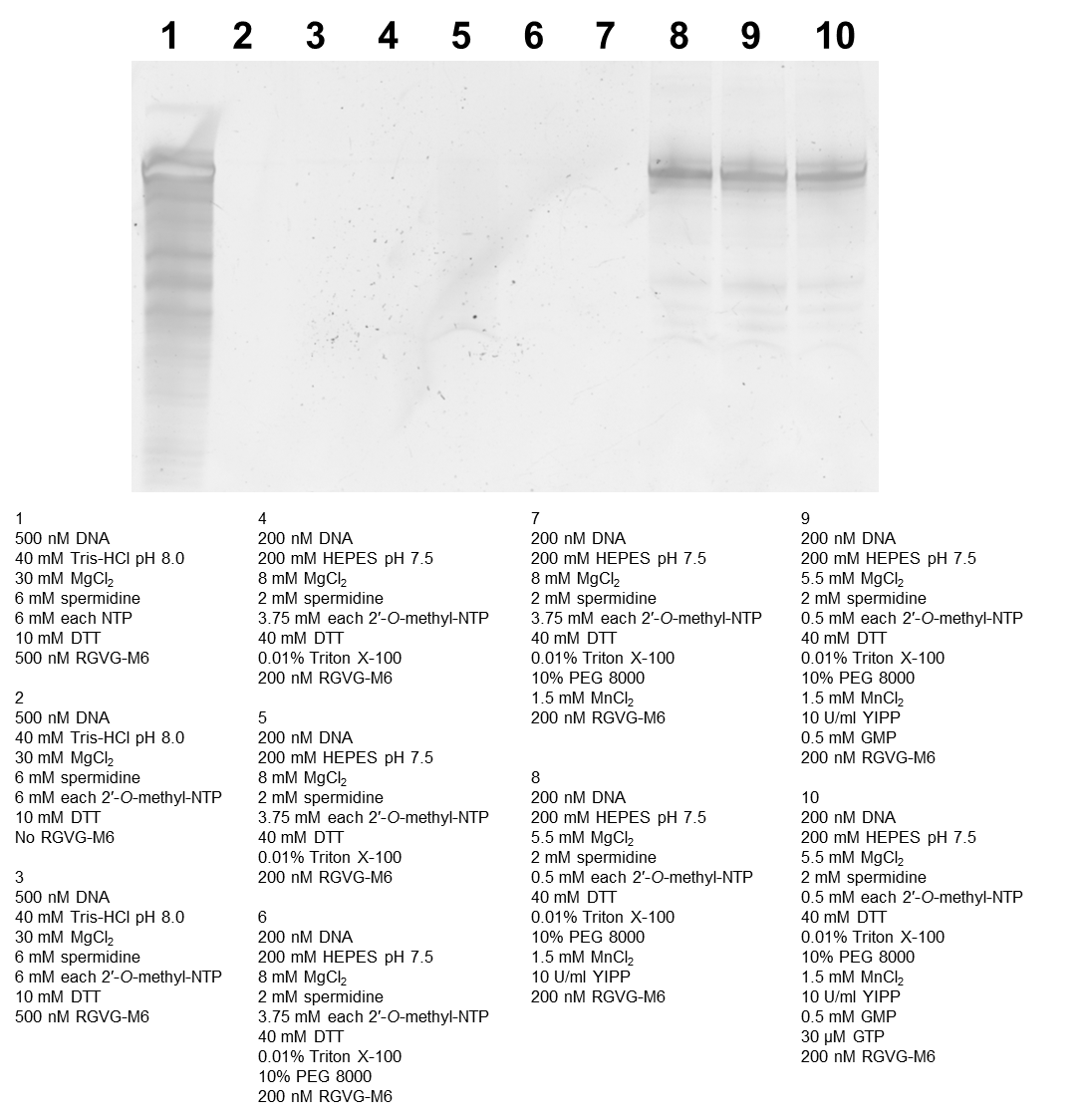
**

**Figure S5. Comparison of RGVG-M5 and RGVG-M6 to Y639L H784A in the transcription of 2′-*O*-methylnucleotides (mN) in permissive buffer (200 mM HEPES pH 7.5, 5.5 mM MgCl_2_, 2 mM spermidine, 0.5 mM each 2′*-O-*methyl-NTP, 40 mM DTT, 0.01% Triton, 10% PEG8000, 1.5 mM MnCl_2_, 10 U/ml yeast inorganic pyrophosphatase, 200 nM RNA polymerase, and 200 nM DNA). Transcripts were analyzed by denaturing PAGE and imaged after staining in SYBR-Gold. Transcriptions ran 20 hours. A reaction containing WT T7 RNA polymerase with ribonucleotides (rN) is shown for comparison.**

**
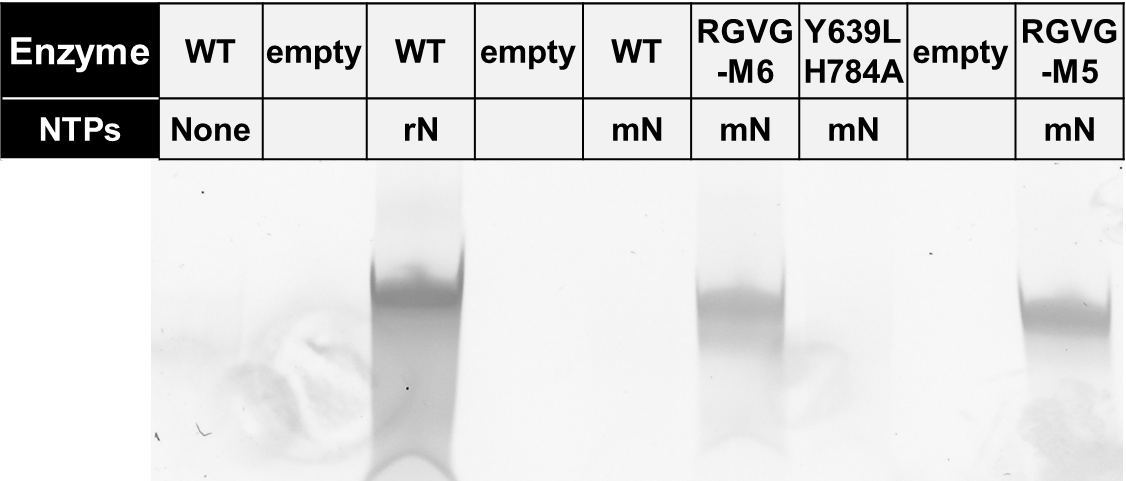
**
